# Hypersensitivity to non-steroidal anti-inflammatory drugs in pediatric patients

**DOI:** 10.1186/1939-4551-8-S1-A120

**Published:** 2015-04-08

**Authors:** Alex Lacerda, Luis Felipe Ensina, Ligia Maria Oliveira Machado, Dirceu Sole, Inês Cristina Camelo Nunes, Djanira Andrade

**Affiliations:** 1 Federal University of Sao Paulo, Brazil; 2 Brazilian Society, Brazil; 3 Federal Universtity of Sao Paulo, Brazil

## Background

Non steroidal anti inflammatory drugs are the main cause of hipersensitivity reactions in Brazil. The aim of this study is to analyze the characteristics of pediatric patients with a suggestive history of hypersensitivity to non-steroidal anti-inflammatory drugs (NSAIDs) in a specialized center.

## Methods

Retrospective study of medical records based on a specific questionnaire adapted from ENDA in patients under 18 years, followed from July 2011 to July 2014.

## Results

Medical records of 104 individuals with a history of hypersensitivity to drugs were analyzed. NSAIDs were the primarily suspected drug in 50% of the cases. The mean age was 10.9 years, with a predominance of males (57.7%). All patients had cutaneous manifestations and isolated angioedema was observed in the majority (53.8%); respiratory symptoms were reported in 44% of cases and gastrointestinal manifestations in 7.6%. Most of the patients reported reactions to more than one NSAID (71%) and dipyrone was the main drug reported in this group (34%), as in the group who had reactions to a single NSAID (73%). All reactions occurred in less than 24 hours after the drug use and 69.2% were of moderate severity. The ER was the main site searched for the treatment of the reactions (88%). During diagnostic investigation 38 oral provocation tests were performed, with positive results in 8 (5 ASA, 1 Ibuprofen, 1 Acetaminophen, 1 Dipyrone).

## Conclusions

The reactions to NSAIDs in pediatric patients are more frequent in males. Angioedema alone was the main clinical manifestation and dipyrone the main drug involved in the reactions. The OPT proved to be an important tool to confirm or exclude the diagnosis of hypersensitivity, with the possibility of providing safer alternatives for those patients with proven reaction.

